# Modulating redox metabolism to improve isobutanol production in *Shimwellia blattae*

**DOI:** 10.1186/s13068-020-01862-1

**Published:** 2021-01-06

**Authors:** Miguel G. Acedos, Isabel de la Torre, Victoria E. Santos, Félix García-Ochoa, José L. García, Beatriz Galán

**Affiliations:** 1 Chemical and Materials Engineering Department, Chemical Sciences School, Universidad Complutense de Madrid, 28040 Madrid, Spain; 2 Department of Microbial and Plant Biotechnology, Centro de Investigaciones Biológicas, CSIC, 28040 Madrid, Spain

**Keywords:** Isobutanol, Redox balance, *Shimwellia blattae*, Synthetic pathway

## Abstract

**Background:**

Isobutanol is a candidate to replace gasoline from fossil resources. This higher alcohol can be produced from sugars using genetically modified microorganisms. *Shimwellia blattae* (p424IbPSO) is a robust strain resistant to high concentration of isobutanol that can achieve a high production rate of this alcohol. Nevertheless, this strain, like most strains developed for isobutanol production, has some limitations in its metabolic pathway. Isobutanol production under anaerobic conditions leads to a depletion of NADPH, which is necessary for two enzymes in the metabolic pathway. In this work, two independent approaches have been studied to mitigate the co-substrates imbalance: (i) using a NADH-dependent alcohol dehydrogenase to reduce the NADPH dependence of the pathway and (ii) using a transhydrogenase to increase NADPH level.

**Results:**

The addition of the NADH-dependent alcohol dehydrogenase from *Lactococcus lactis* (AdhA) to *S. blattae* (p424IbPSO) resulted in a 19.3% higher isobutanol production. The recombinant strain *S. blattae* (p424IbPSO, pIZpntAB) harboring the PntAB transhydrogenase produced 39.0% more isobutanol than the original strain, reaching 5.98 g L^−1^ of isobutanol. In both strains, we observed a significant decrease in the yields of by-products such as lactic acid or ethanol.

**Conclusions:**

The isobutanol biosynthesis pathway in *S. blattae* (p424IbPSO) uses the endogenous NADPH-dependent alcohol dehydrogenase YqhD to complete the pathway. The addition of NADH-dependent AdhA leads to a reduction in the consumption of NADPH that is a bottleneck of the pathway. The higher consumption of NADH by AdhA reduces the availability of NADH required for the transformation of pyruvate into lactic acid and ethanol. On the other hand, the expression of PntAB from *E. coli* increases the availability of NADPH for IlvC and YqhD and at the same time reduces the availability of NADH and thus, the production of lactic acid and ethanol. In this work it is shown how the expression of AdhA and PntAB enzymes in *Shimwellia blattae* increases yield from 11.9% to 14.4% and 16.4%, respectively.

## Background

Nowadays, developed societies have experienced a great increase in the demand for fossil fuels, due to the increase in energy requirements, the growth of the world population and the standards of living [1, 2]. The depletion of non-renewable sources and their environmental impact has made this problem one of the biggest concerns of the present society [3]. Renewable alternatives to conventional fuels derived from petroleum can be developed by engineering microorganisms as biocatalysts for the conversion of renewable feedstocks into petrochemical replacements. Likewise, there is currently a growing interest in obtaining substitutes for bioethanol as biofuel, because this alcohol has a number of limitations [4, 5]. Compared to ethanol, isobutanol offers many advantages both as a substitute for gasoline and improving the properties of gasoline in blends due to its higher energy content and higher hydrophobicity [6, 7]. Isobutanol can also be used as chemical platform to obtain other products with added value [8].

**Fig. 1:**
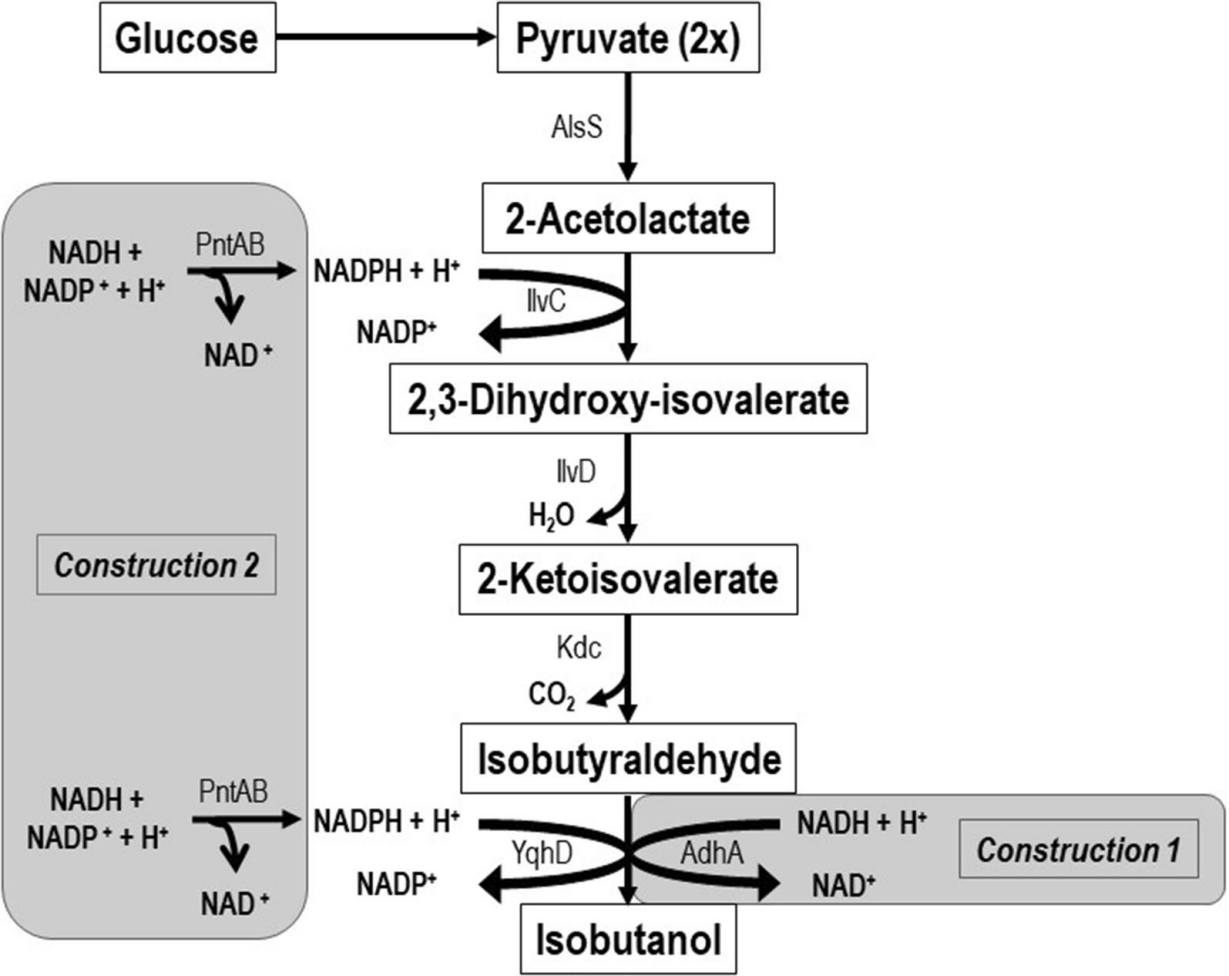
Metabolic pathway for isobutanol production in *S. blattae* (p424IbPSO) and new constructions (1 and 2). Pyruvate (2x) means that two pyruvate molecules are required to synthesize one 2-acetolactate molecule; AlsS, acetolactate synthase; IlvC, acetohydroxy acid isomeroreductase, IlvD, dihydroxyacid dehydratase; Kdc, 2-ketoacid decarboxylase; YqhD, alcohol dehydrogenase (endogenous). In new construction 1, AdhA, alcohol dehydrogenase (*L. lactis*) and in new construction 2, PntAB, nicotinamide nucleotide transhydrogenase (*E. coli*)

**Fig. 2:**
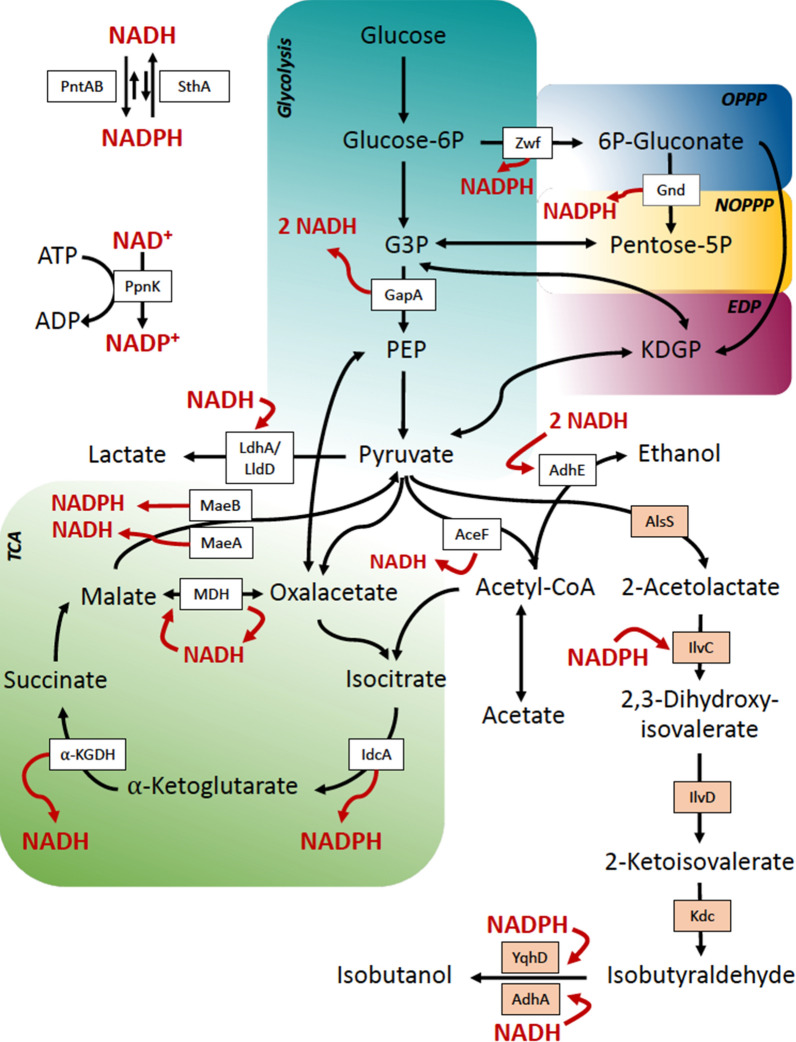
Main metabolic pathways in *S. blattae*, glycolysis, oxidative pentose phosphate pathway (OPPP), non-oxidative pentose phosphate pathway (NOPPP), Entner–Doudoroff pathway (EDP), and tricarboxylic acid (TCA) cycle. PTS, glucose phosphotransferase system; Zwf, glucose-6-phosphate dehydrogenase; Gnd, 6-phosphogluconate dehydrogenase; KDGP, 2-keto-3-deoxy-6-phosphogluconate; G3P, glyceraldehyde-3-phosphate; GapA, glyceraldehyde-3 phosphate dehydrogenases; AceF, pyruvate dehydrogenase; MaeA, NAD-dependent malic enzyme; MaeB, NADP-dependent malic enzyme; MDH, malate dehydrogenase; Icd, isocitrate dehydrogenase; α-KGDH, α-ketoglutarate dehydrogenase; PntAB, membrane-bound transhydrogenase; SthA, soluble transhydrogenase; PpnK, NAD kinase; PEP, phosphoenolpyruvate.; AdhE, aldehyde-alcohol dehydrogenase; IdhA, D-lactate dehydrogenase; IldD, L-lactate dehydrogenase

Several genetically modified microorganisms for isobutanol production have been engineered, including *Escherichia coli* [7, 9–11], *Corynebacterium glutamicum* [12–14], *Saccharomyces cerevisiae* [15–17], *Bacillus subtilis* [14, 18, 19], *Clostridium cellulolyticum* [20], *Clostridium thermocellum* [21] and *Shimwellia blattae* [22], among others. To achieve isobutanol production from glucose the most common strategy has been the derivation of intermediates from amino acid biosynthesis pathways to alcohol production. Most of the modifications carried out are based on the last two steps in the Ehrlich pathway for 2-keto acid degradation and the valine biosynthesis pathway. 2-Keto acids then are converted to aldehydes by heterologous broad-substrate-range 2-keto-acid decarboxylases (KDCs) and then to alcohols by alcohol dehydrogenases (ADHs) [7, 9]. The main limitations of this route are the NADPH/NADH imbalance [18, 23, 24] and the synthesis of by-products [25] (Figs. 1 and 2).

Different strategies have been carried out to modulate the redox metabolism with the aim of avoiding the depletion of reduced co-substrates [26]. In this sense, it has been proposed to perform two genetic modifications in the *E. coli* isobutanol producers, i.e., the overexpression of the PntAB transhydrogenase and the construction of an NADH-dependent isobutanol pathway by changing the dependence of ketol-acid reductoisomerase (IlvC) from NADPH to NADH [27]. The strain was additionally improved through the insertion of the AdhA NADH-dependent alcohol dehydrogenase from *Lactococcus lactis* to circumvent the use of YqhD NADPH-dependent aldehyde reductase of *E. coli* [27]. The improvement in the isobutanol production due to the insertion of the *adhA* gene from *L. lactis* was also proposed in previous works [10, 28]. More recently, Wu et al. [29] has developed a combined approach by modifying the specificity of IlvC and YqhD from NADPH to NADH.

The overexpression of a transhydrogenase enzyme has been also used for the production of some chiral alcohols [30]. The positive effect of PntAB was also observed in an isobutanol producer strain of *C. glutamicum* [13]. Moreover, they observed that the inactivation of the malic enzyme in this strain reduced the yield of isobutanol, since this enzyme is responsible for the conversion of NADH to NADPH [13] (Fig. 2).

The cellular redox state in *E. coli* can be also modulated by inserting a *gapN* gene (glyceraldehyde-3-phosphate dehydrogenase) that reduces NADP to NADPH [24]. The effect of modulating the redox state was also studied in *Saccharomyces cerevisiae*, where the isobutanol production was increased by overexpressing the pyruvate carboxylase, malate dehydrogenase and malic enzyme [17].

Smith et al. [12] have unsuccessfully tried to increase NADPH availability in *C. glutamicum* for the production of isobutanol by inactivating the gene encoding the glucose 6-phosphate isomerase to redirect the carbon flux into the pentose phosphate pathway to generate NADPH.

Previously, we have engineered a synthetic inducible operon (IbPSO) expressing *alsS*, *ilvC*, *ilvD* and *kdc* gene in a wide host range plasmid to produce isobutanol in different bacteria [22, 31]. Using this approach, we selected *S. blattae* as cell factory because it is a very robust host that resists high concentrations of isobutanol and is capable of growing in toxic lignocellulosic wastes [22]. Moreover, this microorganism has been already used for the production of other similar value-added compounds such as 1,3-propanediol [32–35]. In addition, the wild type strain of *S. blattae* only harboring the IbPSO operon was able to produce amounts of isobutanol similar to those produced by other strains that have been extensively engineered and mutagenized, suggesting that this strain could be a promising chassis to attempt a further rational genetic improvement for isobutanol production.

In this study, we considered to investigate whether the influence of the redox balance in the production of isobutanol could render not only a better isobutanol producer but also new insights in this field.

Therefore, the main objective of this work was to improve the production of isobutanol in *S. blattae* (p424IbPSO), following two main strategies independently. First, we have overexpressed the NAD-dependent alcohol dehydrogenase (AdhA) from *L. lactis* to offer a new enzyme to finish the pathway. Second, we have modulated the cell redox balance of the host by cloning the *pntAB* transhydrogenase encoding gene from *E. coli* to increase the NADPH levels required by IlvC and YqhD reductases.

## Results

### Testing the effect of AdhA from *L. lactis* in isobutanol production

To determine the effect of the AdhA aldehyde reductase from *L. lactis* in the production of isobutanol by *S. blattae* (p424IbPSO), we have cloned the *adhA* gene in the wide host range compatible expression vector pIZ2 to construct pIZadhA and transformed *S. blattae* (p424IbPSO) to render the new recombinant strain *S. blattae* (p424IbPSO, pIZadhA) (Table 2).

**Fig. 3:**
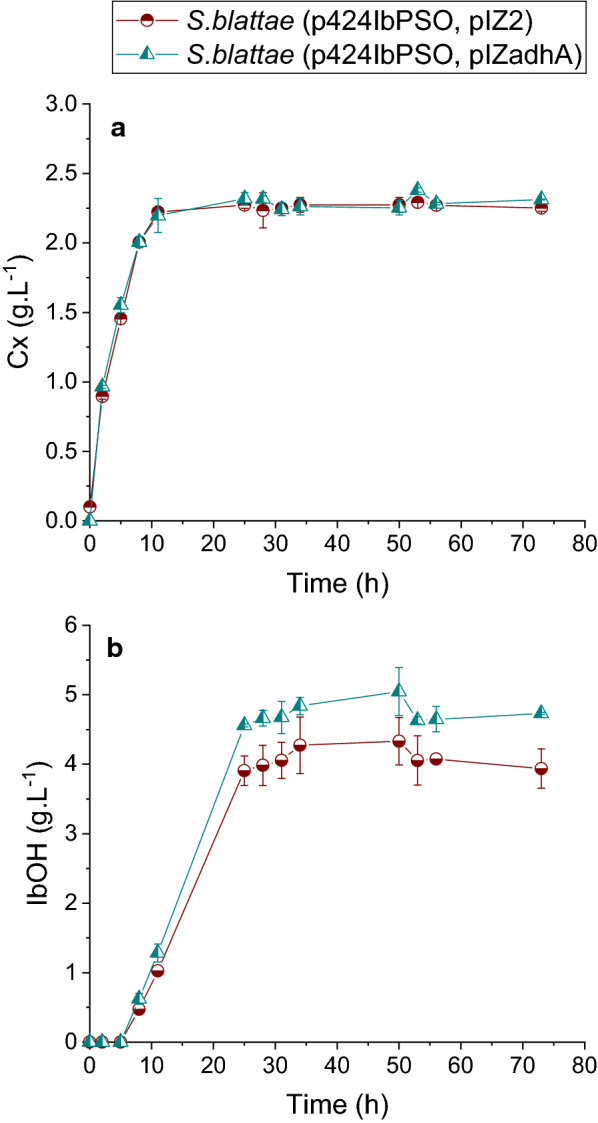
Time course of cell growth (**a**) and isobutanol (**b**), for *S. blattae* (p424IbPSO, pIZ2) and *S. blattae* (p424IbPSO, pIZadhA). These experiments were carried out in sealed bottles. In the figure the term “Cx” refers to biomass concentration expressed in gram per liter and “IbOH” refers to isobutanol concentration expressed in gram per liter. Error bars indicate ± SD (*n* = 3)

*S. blattae* (p424IbPSO, pIZ2) and *S. blattae* (p424IbPSO, pIZadhA) grow similarly in the isobutanol producing medium (Fig. 3a) but the strain carrying the *adhA* gene produced 19.3% more isobutanol (Fig. 3b). This increase in the isobutanol production can be due to the combination of several factors, i. e., increase of isobutyraldehyde reductase activity, reduction of NADPH dependence of the pathway as well as reduction of by-products. In this sense, the new recombinant strain produced 18.6% and 48.3% less lactate and ethanol, respectively, than the strain harboring the empty pIZ2 plasmid (Fig. 5). This result suggests that the NADH-dependent AdhA of *L. lactis* is competing for the NADH pool to transform the overproduced isobutyraldehyde into isobutanol with the NADH-dependent LdhA and AdhE enzymes of *S. blattae* which transform pyruvate and acetyl-CoA into lactate and ethanol, respectively. In the absence of AdhA, isobutanol production is limited to the NADPH pool used by YqhD and thus, LdhA and AdhE enzymes can use the available NADH pool to produce large amounts of lactate and ethanol. Table 1 shows that the productivity, specific production and specific productivity values are higher in *S. blattae* (p424IbPSO, pIZadhA) expressing AdhA than in the control strain.

**Table 1:** Isobutanol productivity, specific production and specific productivity for the three strains constructed and tested in this work

Strain	Productivity (g_IbOH_ L^−1^ h^−1^)	Specific production (g_IbOH_ g_X_^−1^)	Specific productivity (g_IbOH_ g_X_^−1^ h^−1^)
*S. blattae* (p424IbPSO, pIZ2)	0.14 ± 0.008	1.86 ± 0.09	0.06 ± 0.004
*S. blattae* (p424IbPSO, pIZadhA)	0.16 ± 0.007	2.13 ± 0,06	0.07 ± 0.003
*S. blattae* (p424IbPSO, pIZpntAB)	0.19 ± 0.010	2.32 ± 0,12	0.08 ± 0.006

In the table the term “*g*” refers to gram, “*L*” refers to liter, “*h*” refers to hour and “*X*” refers to biomass. Experimental error is indicated as ± SD (*n* = 3)

### Testing the effect of PntAB from *E. coli* in isobutanol production

**Fig. 4:**
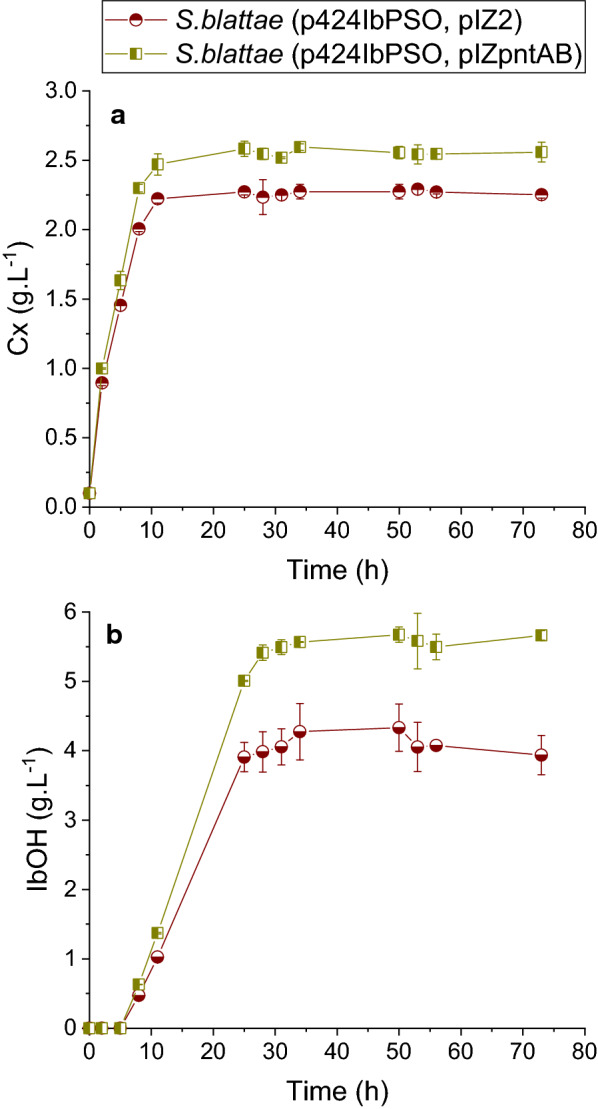
Time course of cell growth (**a**) and isobutanol (**b**) for *S. blattae* (p424IbPSO, pIZ2) and *S. blattae* (p424IbPSO, pIZpntAB). These experiments were carried out in sealed bottles. In the figure the term “Cx” refers to biomass concentration expressed in gram per liter and “IbOH” refers to isobutanol concentration expressed in gram per liter. Error bars indicate ± SD (*n* = 3)

**Fig. 5:**
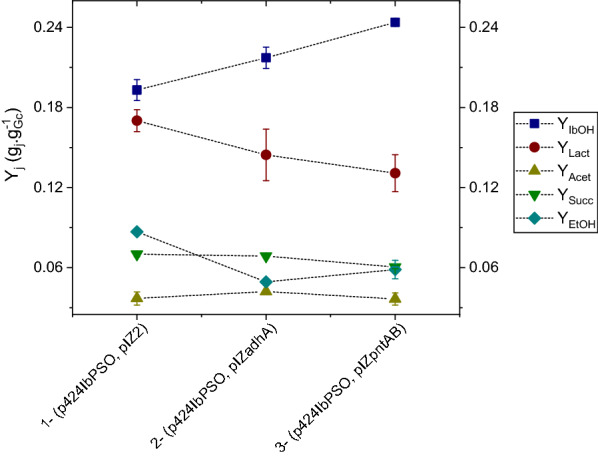
Yield for each metabolite in each of tested strains (Yj). Error bars indicate ± SD (*n* = 3). In the figure: GC, consumed glucose, IbOH, isobutanol; Lact, lactic acid; Acet, acetic acid; Succ, succinic acid and EtOH, ethanol

To determine the effect of the PntAB membrane-integral nicotinamide nucleotide transhydrogenase from *E. coli* in the production of isobutanol by *S. blattae* (p424IbPSO), we cloned the *pntAB* genes in the pIZ2 vector to construct pIZpntAB and transformed *S. blattae* (p424IbPSO) to render the new recombinant strain *S. blattae* (p424IbPSO, pIZpntAB) (Table 2). Interestingly, the strain overproducing PntAB grows slightly better than *S. blattae* (p424IbPSO) (Fig. 4a) and increased the isobutanol titer from 4.30 g L^−1^ to 5.98 g L^−1^, producing 39.0% more isobutanol (Fig. 4b). The isobutanol increase could be due to the combination of two factors, i.e., increase of the IlvC and YqhD reductase activities due to the increase of NADPH levels or to the reduction of by-products. In this sense, the strain produced 24.4% and 31.0% less lactate and ethanol, respectively, than the strain harboring the empty pIZ2 plasmid (Fig. 5). This result suggests that the reduction of the NADH pool in favor of the NADPH pool by PntAB reduces the activity of LdhA and AdhE enzymes of *S. blattae* and, consequently, the production of lactate and ethanol is decreased. The strong reduction of LdhA activity also favors the production of isobutanol since more pyruvate can be channeled to 2-acetolactate by AlsS acetolactate synthase. The increased biomass observed in the strain *S. blattae* (p424IbPSO, pIZpntAB) can be explained because the cellular stress produced by the high consumption of NADPH by the synthetic pathway is partially reduced by the increased level of NADPH generated by PntAB. Table 1 shows that the productivity, specific production and specific productivity values are higher in *S. blattae* (p424IbPSO, pIZpntAB) expressing PntAB than in the other strains constructed up to now.

**Table 2:** Strains, genotype and plasmid used in the experimental work in this study

Strains	Genotype	References
*Shimwellia blattae*	Type strain	CIP 104942, DSMZ 4481
*Escherichia coli* DH5α	*endA1, hsdR17, supE44, thi-1 recA1, gyrA* (Nal^R^), *relA1* Δ*(argF-lac)*, U169 *depR,* Φ*80dlacd(lacZ)* M15	[44]
*Lactococcus lactis* IL1403	Type strain	Provided by Dr. P. López (CIB-CSIC)

Plasmids	Genotype	References
pIZ1016	Gm^R^, broad host range expression vector bearing *lacI*^*q*^ and *Ptac. Rep* (pBBR1MCS)	[46]
pIZ2	Extended pIZ1016 polylinker. *Eco*RI*, Cla*I*, Sca*I*, Sma*I *(Xma*I*), Spe*I*, Xva*I*, Sal*I*, Pst*I *(Sbf*I*), Sph*I*, Hin*dIII and *Sac*I	Provided by Dr. G. Durante (CIB-CSIC)
pSEVA424	Sm^R^/Sp^R^, broad host range expression vector bearing *lacI*^*q*^ and *Ptrc. Rep* (RK2 replicative origin. *oriV*-*trfA*)	[47]
p424IbPSO	Sm^R^/Sp^R^. IbPSO operon (*alsS*-*ilvC*-*ilvD-kdc*) into pSEVA424	[22]
pIZadhA	Gm^R^. *adhA* gene of *L. lactis* into pIZ2	This work
pIZpntAB	Gm^R^. *pntAB* gene of *E. coli* into pIZ2	This work

## Discussion

To increase the production of isobutanol in the recombinant strain *S. blattae* (p424IbPSO) we have investigated two different approaches that modify the NADPH/NADH balance of the cell. As it is shown in Fig. 1, *S. blattae* uses NADPH as a co-substrate in two steps of the synthetic metabolic pathway, i.e., the transformation of 2-acetolactate into 2,3-dihydroxyvalerate by IlvC and the transformation of isobutyraldehyde into isobutanol performed by YqhD. Thus, the main limitation of this metabolic pathway occurs when NADPH is exhausted due to a shortage of oxygen, or any other reason that promotes an imbalance of NADPH/NADH co-factors. This bottleneck reduces the production of isobutanol in favor of the production of other undesired metabolites, such as lactate, ethanol or acetate. The importance of the redox state in the production of isobutanol has been demonstrated by other authors. Jung and collaborators [36] showed how the reduction in the consumption of co-factors increases their availability for the isobutanol biosynthetic enzymes, thus increasing alcohol production. Recently, the importance of the NADPH levels in isobutanol production have been demonstrated in cell-free systems showing a correlation between high NADP+ levels and low isobutanol production yields [37]. To reduce this limitation, we have implemented and compared two independent and not synergic solutions. Firstly, we have expressed the AdhA dehydrogenase from *L. lactis* that uses NADH to transform the isobutyraldehyde into isobutanol [10, 27]. This addition produces several benefits including increased isobutanol and decreased lactate and ethanol production, presumably by (i) reducing NADPH dependence of the pathway; (ii) leaving more NADPH free to be used by IlvC; (iii) consuming NADH and reducing its availability for the undesired reductive reactions. The overproduction of AdhA by a multicopy expression plasmid also contributes to this aim. It is interesting to notice that unexpectedly the overexpression of the *adhA* gene does not cause stress in the host cells, since the growth rate and the final biomass obtained are similar for *S. blattae* (p424IbPSO, pIZ2) and *S. blattae* (p424IbPSO, pIZadhA) strains (Fig. 3). In this way, the production of isobutanol was increased up to nearly 20% in the *S. blattae* (p424IbPSO, pIZadhA) strain when compared with the strain without AdhA. This leads to a yield increase of 7.2%, i. e., from 46.4 to 53.7% on the theoretical maximum yield. On the other hand, a large decrease in the yield of secondary metabolites, lactic acid and ethanol is clearly observed (Fig. 5). Since the production of these two by-products requires the consumption of NADH by Ldh and Adh that are NADH-dependant enzymes, the high consumption of the co-substrate by the overproduced AdhA strongly reduces the activity of Ldh and Adh [24]. Most probably, this high consumption of NADH by AdhA is favored by an increase in the availability of its co-substrate, isobutyraldehyde that is now overproduced in the pathway due to the higher accessibility of IlvC for NADPH. These results are consistent with those obtained by other authors who tested different alcohol dehydrogenases in *E. coli* and observed that the overexpression of AdhA from *L. lactis* increased isobutanol production by approximately 1.0 g L^−1^ more than the overexpression of YqhD from *E. coli* [10]. These authors determined the catalytic constants for YqhD and AdhA for the two substrates, acetaldehyde and isobutyraldehyde. Although AdhA has higher affinity and a major reaction rate (k_cat_) to acetaldehyde, it increases the isobutanol production due to the use of the more abundant NADH co-substrate. The results obtained in this work are also compatible with those observed by Bastian et al. [27] that improved the isobutanol yield from 2.1% to 2.6% using AdhA from *L. lactis* under anaerobic conditions. In *S. blattae*, we have improved the total yield from 11.9% to 14.4%.

Secondly, we have tested the possibility to increase the production of isobutanol by unbalancing the NADH/NADPH ratio in favor of NADPH by overexpressing the PntAB transhydrogenase of *E. coli* in *S. blattae* (p424IbPSO). The objective of this approach was to increase the availability of NADPH for IlvC and YqhD enzymes. Unexpectedly, *S. blattae* (p424IbPSO, pIZpntAB) overproducing PntAB yielded more biomass and higher growth rate than the *S. blattae* (p424IbPSO, pIZ2) which does not overexpress *pntAB* (Fig. 4). As the enzymes coded by the synthetic IbPSO operon harbored in p424IbPSO, are NADPH dependent, we speculate that higher availability of NADPH could reduce cell stress, resulting in an increased cell growth [38]. Likewise, isobutanol production is also notably higher in *S. blattae* (p424IbPSO, pIZpntAB) cultures, reaching a production about 2.0 g_IbOH_.L^−1^ more than the reference strain *S. blattae* (p424IbPSO, pIZ2). We assume that the transformation of NADH cellular pool into NADPH increased the pathway flux through the production of isobutanol. It is known that the overproduction of PntAB increased the production of bio-based chemicals where production pathways contain NADPH-dependent enzymes [27, 30, 38]. In our case, the calculated yield over the theoretical maximum increases from 46.4% to 58.5%. On the other hand, the production of NADH-dependent metabolites as by-products (lactic acid and ethanol) is even lower, because of the lower availability of NADH (Fig. 5). As expected, the production of succinic acid as a by-product is not affected by the modifications carried out in this work, because its synthesis does not depend of NADH/NADPH balance. Moreover, we cannot discard that PntAB overproduction can lead to an increased resistance of *S. blattae* (p424IbPSO, pIZpntAB) to alcohols as described in other organisms [39, 40], and although *S. blattae* is a robust strain [41, 42] its growth is inhibited at concentrations of isobutanol above 8 g L^−1^ [22]. This fact can also explain the observed higher growth of *S. blattae* (p424IbPSO, pIZpntAB) (Fig. 4). Our results support other studies in which the overexpression of *pntAB* in *E. coli* improved the isobutanol yield from 2.1% to 9.6% [27] or the regulation of this gene increases 8.0% and 20.0% the production and titer of isobutanol, respectively [43]. In addition, the overexpression of the *pntAB* gene in *B. subtilis* (BSUL09) also increased the concentration of isobutanol by approximately 0.4 g L^−1^ (from 2.2 to 2.6 g.L^−1^) [18]. In our case, overexpression of *pntAB* in *S. blattae* (p424IbPSO) improved the total yield from 11.9% to 16.4%.

## Conclusions

The results presented above allow us to conclude that the availability of NADPH is a major bottleneck, rather than the availability of pyruvate, for the production of isobutanol by the recombinant *S. blattae* strain harboring the plasmid p424IbPSO with the synthetic IbPSO operon. The solution provided by the addition of extra copies of *pntAB* of *E. coli*, appears to be more effective than the utilization of an NADH-dependent step to transform isobutyraldehyde into isobutanol by the addition of the NADH-dependent AdhA dehydrogenase that also increases isobutanol production by 19.3% and isobutanol yield from 11.9% to 14.4%. This result agrees with the observation of Atsumi and collaborators [10] who showed that the overexpression of AdhA increased the isobutanol production about 15% over the previous level of 7.54 g L^−1^ and with Bastian and collaborators [27] who with the same strategy managed to increase the isobutanol yield from 2.1% to 2.6%. This result also agrees with a recent report on cell-free isobutanol production that showed that increasing NADPH concentrations also increased isobutanol titer [37]. However, a further deletion of *ydhD* gene reduced the isobutanol production, suggesting that both enzymes, AdhA and YqhD, can function in a complementary way [12]. Therefore, the option of deleting the *yqhD* in *S. blattae* (p424IbPSO, pIZadhA) in order to increase the availability of NADPH for IlvC was not considered. Interestingly, by the single overexpression the PntAB transhydrogenase, we have been able to increase 39.0% the production of isobutanol in *S. blattae* (p424IbPSO, pIZpntAB) and isobutanol yield from 11.9% to 16.4%. Bastian et al. [27] managed to increase isobutanol yield from 2.1% to 9.1% with the same strategy and also the expression of this gene increases about 8.0% the production of isobutanol [43]. A combined overexpression of AdhA and PtnAB would not be synergistic or additive, since the presence of AdhA and PntAB in the same strain will work in opposite directions, since the overproduction of AdhA reduces the availability of NADH for PntAB, and the presence of PntAB reduces the availability of NADH for AdhA. Finally, we have to mention that the option of combining the overexpression of AdhA with a NADH-dependent IlvC has not been tested, but we assume that according to the results of Bastian et al. [27] and Wu et al. [29], we anticipate that the isobutanol production will not increase beyond the 39.0% achieved by *S. blattae* (p424IbPSO, pIZpntAB).

## Methods

### Bacterial strains, plasmids, growth media and culture conditions

The bacterial strains and plasmids used in this study are listed in Table 2. *E. coli* DH5α and *S. blattae* strains were cultured in solid LB medium at 37 °C. LB medium (solid and broth) was employed for bacterial propagation as described [22]. Antibiotics were used for plasmid maintenance if indicated at the following concentrations: gentamicin (10 µg mL^−1^) and streptomycin (50 µg mL^−1^). The recombinant strains constructed in this work were cultured in M92X minimal liquid media containing a mixture of trace elements (nitrilotriacetic acid (1.5 mg L^−1^), MgSO_4_·7H_2_O (3 mg L^−1^), ZnSO_4_·7H_2_O (0.18 mg L^−1^), CuSO_4_·5H_2_O (0.01 mg L^−1^), MnSO_4_·2H_2_O (0.5 mg L^−1^), NaCl (1 mg L^−1^), FeSO_4_·7H_2_O (0.1 mg L^−1^), CoSO_4_·7H_2_O (0.18 mg L^−1^), NaSeO_3_·5H_2_O (0.3 mg L^−1^), KAl(SO_4_)_2_·12H_2_O (0.02 mg L^−1^), H_3_BO_3_ (0.01 mg L^−1^), Na_2_MoO·2H_2_O (0.01 mg L^−1^), NiCl_2_·6H_2_O (0.025 mg L^−1^)) [31]. This medium was supplemented with yeast extract (1.5 g L^−1^) [31]. Glucose (20 g L^−1^) was used as carbon source to create *S. blattae* inoculum in minimal medium. *L. lactis* was grown on LB media at 30 °C in an orbital shaker at 200 rpm to extract genomic DNA.

To produce isobutanol we used M92X medium [31], supplemented with yeast extract (1.5 g L^−1^) and glucose (36 g L^−1^) as carbon sources. IPTG at a final concentration of 0.5 mM was added to culture medium for IbPSO operon induction [22]. The pre-inoculum and inoculum steps were performed in order to obtain cells in the same metabolic state to improve the reproducibility of results. Pre-inoculum and inoculum were carried out in 250-mL flasks containing 50 mL of M92X at 37 ℃ and 250 rpm in an orbital shaker, during 12 h and 3 h, respectively. The isobutanol production was performed in sealed bottles in an orbital shaker [22]. The culture was started at 37 ℃, 250 rpm and in aerobic conditions. IPTG was added to the medium after 2 h of incubation for inducing the expression of the operon and the temperature and agitation were reduced to 30 ℃ and 200 rpm, respectively, and the bottles were also closed to avoid the loss of isobutanol by evaporation.

### DNA manipulations and sequencing

DNA manipulations and other molecular biology techniques were essentially as described by Sambrook and Russell [44]. Isolation of *L. lactis* and *E. coli* MG1655 genomic DNA was performed with the Bacteria Genomic Prep Mini Spin Kit (GE Healthcare). Oligonucleotides were purchased from Sigma-Aldrich. DNA amplification was performed on a Mastercycler Gradient (Eppendorf) using DNA polymerase I and *Pfu* polymerase from Biotools B. M. Labs. Reaction mixtures contained 1.5 mM MgCl_2_ and 0.25 mM dNTPs. DNA fragments were purified with High Pure PCR System Product Purification Kit (Roche). Restriction enzymes were obtained from various suppliers and were used according to their specifications. Plasmid DNA was prepared with a High Pure Plasmid Isolation Kit (Roche Applied Science). *Escherichia coli* and *S. blattae* were transformed by electroporation [45]. All cloned inserts and DNA fragments were confirmed by DNA sequencing through an ABI Prism 377 automated DNA sequencer (Applied Biosystems Inc.) at Secugen S.L.

### Construction of plasmids pIZadhA and pIZpntAB

The primers used in this work are shown in Table 3. The *adhA* gene was PCR amplified from *L. lactis* genomic DNA using primers ADH-F 5´-CCCCCCGGGTGACTAAGGAGGTGAATAATGAAAGCAGCAGTAGTAAGACAC-3 (the sequence corresponding to the engineered *Xma*I site is underlined) and ADH-R 5´-GCTCTAGATTATTTAGTAAAATCAATGACCATTCGGC -3´ (the sequence corresponding to the engineered *Xba*I site is underlined). The resulting 1,023-kb DNA fragment was digested with *Xma*I and *Xba*I and cloned into the double-digested *Xma*I and *Xba*I pIZ2 vector to produce plasmid pIZadhA. This plasmid was transformed into *E. coli* DH5α to generate the recombinant strain *E. coli* DH5α (pIZadhA). The plasmid pIZadhA isolated from *E. coli* and transformed *S. blattae* (p424IbPSO)-competent cells yielded *S. blattae* (p424IbPSO, pIZadhA) recombinant strain.

**Table 3:** Primers employed for DNA amplification in this study

Name	Sequence (5´–3´)
ADH-F	CCCCCCGGGTGACTAAGGAGGTGAATAATGAAAGCAGCAGTAGTAAGACAC (*Xm*aI)
ADH-R	GCTCTAGATTATTTAGTAAAATCAATGACCATTCGGC (*Xba*I)
PntAB-F	CGCTGCAGTCATCAATAAAACCGATGGAAGGG (*Pst*I)
PntAB-R	CGAGCTCAGCAGAGGCCGTCAGGG (*Sac*I)

The *pntAB* genes were PCR amplified from *E. coli* MG1655 genomic DNA using primers PntAB-F 5´-CGCTGCAGTCATCAATAAAACCGATGGAAGGG-3´ (the sequence corresponding to the engineered *Pst*I site is underlined) and PntAB-R 5´-CGAGCTCAGCAGAGGCCGTCAGGG-3´ (the sequence corresponding to the engineered *Sac*I site is underlined). The resulting 3,021-kb DNA fragment was digested with *Pst*I and *Sac*I and cloned into the double-digested *Pst*I and *Sa*cI pIZ2 vector to produce plasmid pIZpntAB. This plasmid was transformed into *E. coli* DH5α to generate the recombinant strain *E. coli* DH5α (pIZpntAB). The plasmid pIZpntAB isolated from *E. coli* and transformed *S. blattae* (p424IbPSO)-competent cells yielded *S. blattae* (p424IbPSO, pIZpntAB) recombinant strain.

### Analytical methods

The biomass concentration was determined by measuring the optical density of the cultures at 600 nm in a spectrophotometer (Shimadzu UV–VIS, Japan). The relation of dry cell mass concentration (Cx) and absorbance at 600 nm was obtained by drying biomass at 105 °C until constant weight, and OD at 600 nm is given by the following equation:

$$C_{X} \left( \frac{g}{L} \right) = OD_{600nm} 0.489.$$

The concentrations of glucose, isobutanol and other metabolites were measured by HPLC (Agilent Technologies, 1100 series). A refraction index detector (RID) and HPLC column Rezex RHM-Monosaccharide-H^+^ 300 × 7.8 mm column (Phenomenex) was used in this work. A solution of 1 mM H_2_SO_4_ was employed as mobile phase at a flow rate of 0.5 mL min^−1^. The column temperature was maintained at 80 ºC.

## Calculations

The yields of the products were determined according to the following equation:

$$Y_{J} \left( {\frac{{g_{J} }}{{g_{{{\text{gluc.cons}}}} }}} \right) = \frac{{C_{J} }}{{C_{{{\text{gluc.cons}}}} }}\left( {J = {\text{IbOH, EtOH, Ace, Lac, Succ}}} \right).$$

In the equation, the term “*g*” refers to gram, “*C*” refers to concentration, and term "*j*" refers to compound “gluc” (glucose), “gluc.cons” (glucose consumed), “IbOH” (isobutanol), “EtOH (ethanol), “Ace” (acetic acid), “Lac” (lactic acid) and “Succ” (succinic acid).

Theoretical maximum yield (TMY) of the process was determined as previously described [7]. The TMY value for the isobutanol production is 0.41 g_IbOH_.g_gluc.cons_^−1^.

The productivity (*P*) of isobutanol (IbOH) was determined according the following equation:

$$P_{{{\text{IbOH}}}} \left( {\frac{{g_{{{\text{IbOH}}}} }}{L.h}} \right) = \frac{{C_{{{\text{IbOH}}}} }}{t}.$$

The specific production of isobutanol (SP_IbOH_) was determined as follows:

$$SP_{{{\text{IbOH}}}} \left( {\frac{{g_{{{\text{IbOH}}}} }}{{g_{X} }}} \right) = \frac{{C_{{{\text{IbOH}}}} }}{{C_{X} }}.$$

The term “*X*” refers to biomass.

The specific productivity of isobutanol (SPX_IbOH_) was determined as follows:

$$SPX_{{{\text{IbOH}}}} \left( {\frac{{g_{{{\text{IbOH}}}} }}{{g_{X} .h}}} \right) = \frac{{{\raise0.7ex\hbox{${C_{IbOH} }$} \!\mathord{\left/ {\vphantom {{C_{IbOH} } {C_{X} }}}\right.\kern-\nulldelimiterspace} \!\lower0.7ex\hbox{${C_{X} }$}}}}{t}.$$

## Data Availability

All data obtained during this work are included in this published article.
